# Aiweixin, a traditional Uyghur medicinal formula, protects against chromium toxicity in *caenorhabditis elegans*

**DOI:** 10.1186/s12906-015-0783-4

**Published:** 2015-08-18

**Authors:** Binggen Zhu, Ping Yang, Nurahmat Mammat, Hui Ding, Junmin He, Yong Qian, Jian Fei, Kaiser Abdukerim

**Affiliations:** College of Xinjiang Uyghur Medicine, Hetian, Xinjiang 848000 China; Tongji University School of Medicine, Shanghai, 200092 China; Shanghai Research Center for Model Organisms, Shanghai, 201203 China; Shanghai Standard Biotech Co. Ltd, Shanghai, 201203 China; School of Life Sciences and Technology, Tongji University, Shanghai, 200092 China

**Keywords:** Aiweixin, Traditional Uyghur Medicine, Chromium Toxicity, *Caenorhabditis elegans*

## Abstract

**Background:**

Aiweixin (AWX) is a traditional Uyghur medicine prescription, and has been mainly used to treat heart and brain diseases for a long time. Previous studies indicated that AWX had therapeutic effects in a rat model of myocardial ischemia reperfusion injury. In this study, we investigate whether AWX has protective effects against chromium toxicity in *Caenorhabditis elegans* (*C. elegans*).

**Methods:**

The AWX decoction was the conventional product for clinical use. It was added into M9 buffer in a certain volume for the treatment to the wild-type *C. elegans* and mutational worms, *daf-16, glp-1(notch), daf-2, rsks-1 and eat-2.* Assays for hexavalent chromium {Cr(VI)} stress and reactive oxygen species (ROS) production were used.

**Results:**

We found that AWX at moderate contents (0.083, 0.1, 0.125 volume of AWX/total volume) increased resistance of *C. elegans* to Cr(VI) exposure, although higher contents of AWX are toxic for *C. elegans.* The protective effect of AWX was DAF-16-dependent, but independent on the DAF-2, GLP-1, RSKS-1 and EAT-2. AWX (0.1 volume of AWX/total volume) significantly reduced ROS production of *C. elegans* induced by Cr(VI) exposure.

**Conclusion:**

These results indicated the AWX protected against the toxicity of Cr(VI) in *C. elegans,* and the oxidative stress protective mechanism in worms should be involved.

## Background

Aiweixin (AWX) is a traditional Uyghur medicine prescription and consists of 15 ingredients including *Dracocephalum moldavicum* L., *Eletteria cardamomum* (L.) Maton, *Salix caprea* L. (Salicaceae) flowers, *Lavandula augustifolia* (lavender), *Borago officinalis* L. (Boraginaceae) stems and leaves, *Borago officinalis* L. (Boraginaceae) flower, *Nardostachys jatamansi* DC. root and rhizome (Nardostachyos Radix et Rhizoma), *Bombyx mori* (Abresham) silk cocoons, *Usnea longissima* Ach., *Rosa rugosa* Thunb. flowers, *Syzygium aromaticum* L., *Lindera caudata* (Nees) Hook.f., *Myristica fragrans* (Houtt.), *Crocus sativus* L. and Moschus [1]. AWX is orally administered as a decoction. According to the theory of traditional Uyghur medicine, AWX has preventive and treatment effects in many aspects, including modulating “Mizaj”, balancing body fluid, improving blood circulation, strengthening the function of heart and brain, *etc.* It has been used to treat diseases induced by abnormal “savda”, such as coronary heart disease, myocardial ischemia, arrhythmia, cerebral infarction, depression [1, 2]. The effectiveness of AWX has been well documented during long-term clinical practice. Previous pharmacological experiments demonstrated that AWX exerted therapeutic effects in a rat model of myocardial ischemia reperfusion injury, possibly *via* alleviation of oxidative stress [3, 4].

Chromium exists mostly in two valence states in nature: hexavalent chromium {Cr.(VI)} and trivalent chromium [5]. The trivalent chromium is essential for organisms, and is acknowledged as a dietary supplement. But the Cr(VI) and its compounds have been recognized as having potential severe adverse effects on health. Previous studies demonstrated that Cr(VI) induces oxidative stress, DNA damage, apoptotic cell death and altered gene expression [5–7]. The *Caenorhabditis* elegans (*C. elegans*), a free living nematode that lives mainly in the liquid phase of soils, is considered as an ideal model organism because of its short life span, ease of manipulation, and low cost. It has been found favor as a valuable bioindicator organism in metal toxicity study for its best-characterized properties at the genetic, physiological, molecular, and developmental levels [8, 9]. Assays using *C. elegans* to observe the lethality and sublethal endpoints, growth, reproduction, lifespan, locomotion behavior, stress response, and oxidative damage have been well developed to monitor the toxicity of heavy metals including Cr(VI) [8]. In the present study, we investigate whether AWX has protective effects against Cr(VI) toxicity in *C. elegans*, in order to get more preclinical data which support the therapeutic effects of AWX.

## Methods

### Preparation and phytochemical profile of AWX

The AWX decoction was the conventional product for clinical use, manufactured by Xinwei Pharmaceutical Factory (Hetian, Xinjiang Uyghur Autonomous Region, P. R. China). According to the Pharmacopeia of P. R. China [1], the product is made from *Dracocephalum moldavicum* L. (15 g), *Eletteria cardamomum* (L.) Maton (15 g), *Salix caprea* L. (Salicaceae) flowers (10 g), *Lavandula augustifolia* (lavender) (15 g), *Borago officinalis* L. (Boraginaceae) stems and leaves (10 g), *Borago officinalis* L. (Boraginaceae) flower (10 g), *Nardostachys jatamansi* DC. root and rhizome (Nardostachyos Radix et Rhizoma) (10 g), *Bombyx mori* (Abresham) silk cocoons (50 g), *Usnea longissima* Ach. (3 g), *Rosa rugosa* Thunb. flowers (15 g), *Syzygium aromaticum* L. (15 g), *Lindera caudata* (Nees) Hook.f. (10 g), *Myristica fragrans* (Houtt.) (15 g), *Crocus sativus* L. (0.6 g) and Moschus (0.2 g). The AWX decoction was prepared as follows: before 13 dry herbs were smashed into rude powder, decocted and boiled 3 times (1.5 h for each) while 300 ml of distilled water was collected. The decocted liquid merged the 300 ml of distilled water was filtrated, concentrated, and added with ethanol to 70 % the content of ethanol. The decoction was filtrated again after 24 h while ethanol was collected. Meanwhile, after the two dry medicinal materials was put in 70 % ethanol solution, were extracted by reflux two times (2 h for each). The extracted solution was filtrated while ethanol was collected. The former decoction and the latter extracted solution were mixed and added with distilled water to 1000 ml, then filtrated and sterilized.

**Fig. 1:**
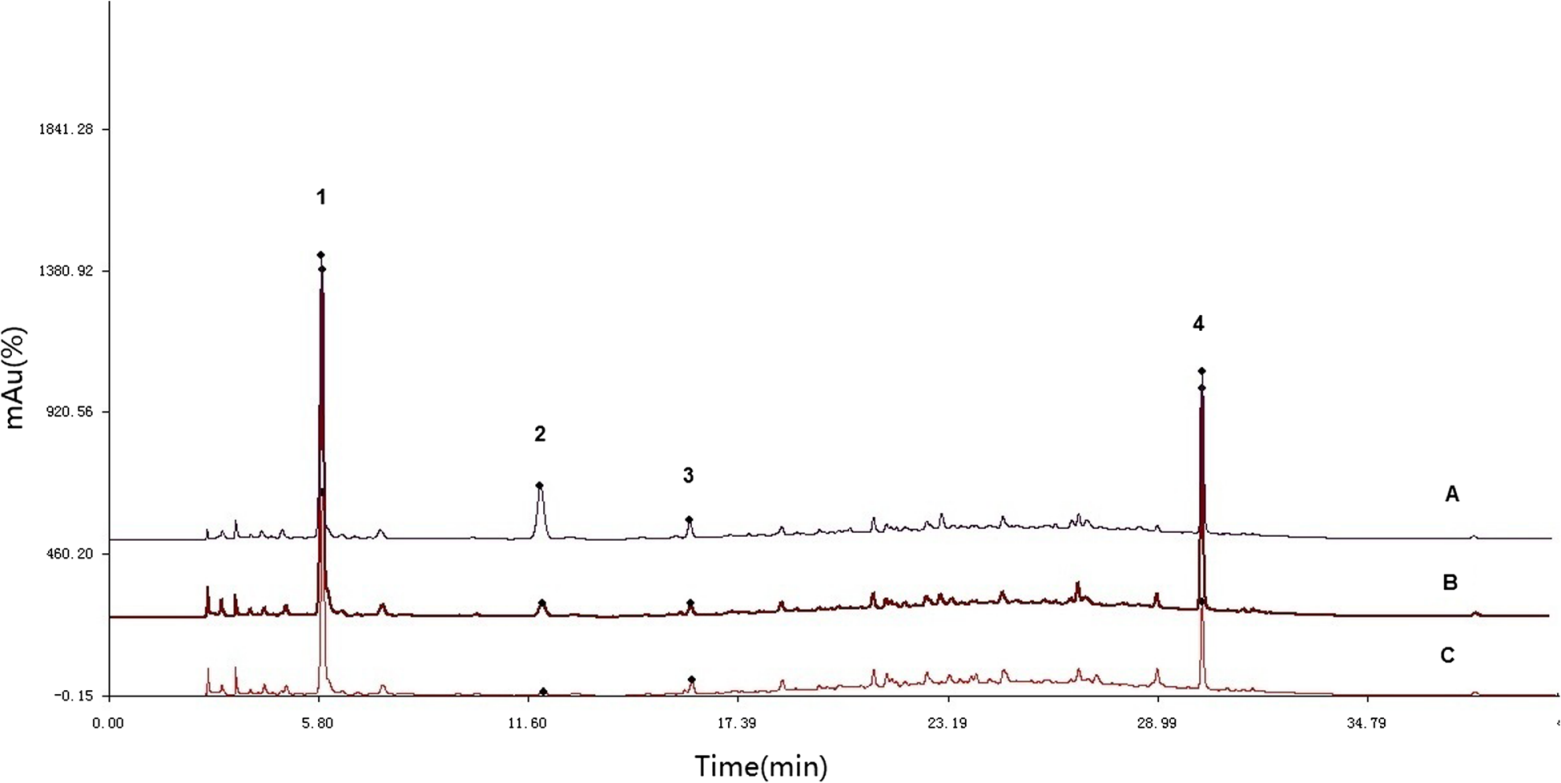
The HPLC fingerprint of three separated AWX samples. A, B and C represented three separated AWX samples

For the quality control of AWX decoction, high-performance liquid chromatography-diode array detection (HPLC-DAD) method has been used, referred to the previous paper [10]. The chromatographic separation was performed by a UItimate XB-C18 (250 mm × 4.6 mm, 5 μm) column in Agilent 1200 (Agilent Technologies). Mobile phase was a gradient of Methanol-acetic acid water gradient. The effluent was monitored on a DAD detector. The fingerprint was set up on the data at 280 nm. The Fig. 1 showed the HPLC fingerprint of three separated AWX samples. By using HPLC–UV-ESI-TOF-MS system, 4 peaks were recognized according to compound molecular weight data. The highest peak 1 contains chebulic acid, while peak 2–4 represent gallic acid, protocatechuic acid, and eugenol respectively. However, others (more than 10 low peaks) were unidentified.

### *Caenorhabditis elegans* strain and maintenance

The wild-type *C. elegans* strain N2 (Bristol) and mutational worms, *daf-16 (mu86), glp-1(e2141), daf-2 (e1370), rsks-1 (ok1255) and eat-2 (ad465)* were provided by the Caenorhabditis Genetics Center (University of Minnesota, Minneapolis, MN). Nematodes were generally incubated at 20 °C on nematode growth media (NGM) plates with E. coli OP50. For culture of *daf-2 (e1370)*, and its wild-type control, nematodes were firstly developed at 16 °C for 3 days and then transferred to 21 °C for the desired stage of development.

### Chromium stress assay and AWX treatment to *C. elegans*

The procedure of chromium stress assay was based on our previous studies [11]. Synchronized Day 3 adult worms were collected and washed with M9 buffer for three times to remove the OP50 bacteria. Approximately 35 adults were suspended into each well of a 48-well culture plate containing 120 μl M9 buffer with or without certain content of AWX (0.05, 0.067, 0.083, 0.1, 0.125 volume of AWX/total volume). 30 min later, K2Cr2O7 {Cr(VI), Sangon Biotech, Shanghai, China} was added at final concentration of 10 mM, except for the experiments without Cr(VI) exposure. The plates were cultivated at 20 °C for certain time. Survived nematodes were counted at different time points. The nematodes were judged to be dead if they did not respond to stimulus using a small, metal wire. Each experiment was reiterated for at least three times.

### Reactive oxygen species (ROS) production

The method of worms received Cr(VI) exposure and treatment of AWX was same as above described in the chromium stress assay. The assay of ROS production was based on published studies [7]. The examined nematodes were transferred to 0.5 mL of M9 buffer containing 5 μM CM-H2DCFDA (Sigma-Aldrich, USA) and pre-incubated for 3 h at 20 °C, and then mounted on 2 % agar pads and examined with a fluorescence microscope (Nikon, SMZ 1500, Japan) at 480/40 nm of excitation wavelength and 535/50 nm of emission filter. Fluorescence levels were measured using Image Software (NIS-Elements D3.1) by determining average pixel intensity in each animal. More than 30 animals were counted for the statistical. The relative fluorescence intensities of the worm were semiquantified. The semi-quantified ROS was expressed as relative fluorescent units (RFU). Three replicates were performed.

### Statistic

The results are reported as mean ± SEM. Significant differences between groups were tested by one-way analysis of variance (ANOVA) followed by Student’s *t*-test. Differences among multiple means were assessed by one-way ANOVA with Bonferroni correction. Statistical software OriginPro 7.5 was used (http://www.originlab.com/). Probability values less than or equal to 0.05 were considered statistically significant.

## Results

### High contents of AWX are toxic for *C. elegans*

**Fig. 2:**
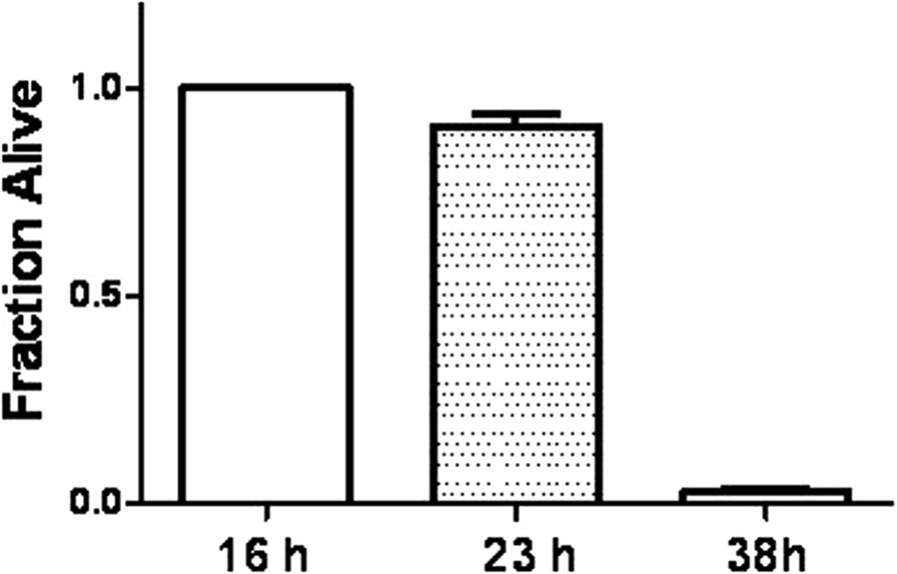
The survival measurement of wild-type *C. elegans* which did not receive any treatments

All of synchronized day 3 adult worms used in our experiments survived well after 16 h incubation in M9 buffer without any additional compositions (seen the Fig. 2). Most (about 91.0 %) of worms were still in good condition after 23 h, but the overwhelming majority was not alive at 38 h incubation. To understand the basic effects of AWX for *C. elegans*, the toxicity and safety of AWX were assessed. As shown in Table 1, the mortality of *C. elegans* was increased with the elevation of AWX contents, after 16 h incubation. AWX at 0.1 volume of AWX/total volume was a little of toxicity (death ratio was 3/34, 8.8 %), but AWX at 0.2 and 0.333 were very toxic {death ratio was 37/46 (80.4 %) and 45/45 (100 %), respectively}. The worms incubated with AWX at 0.067 and 0.083 lived as well as the ones without the treatment of AWX.

**Table 1:** Toxicity and safety of AWX for *C. elegans*

	Contents of AWX (volume of AWX / total volume)					
	0 (control)	0.067	0.083	0.1	0.2	0.333
Survival-16 h	38	49	36	31	9	0
Death-16 h	0	0	0	3	37	45
Total	38	49	36	34	46	45

### AWX increased resistance of *C. elegans* to Cr(VI) exposure in a time and dose-dependent manner

**Fig. 3:**
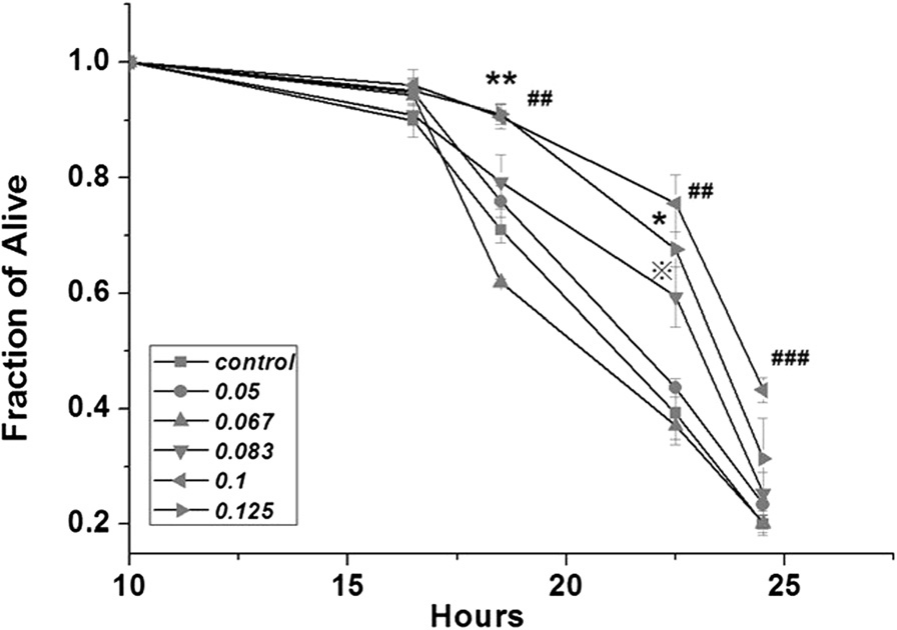
The AWX increased resistance of wild type *C. elegans* to heavy metal Cr(VI) exposure in a time and dose-dependent manner. The X-axis is hours, namely the time of treatment of AWX. The Y-axis is fraction of alive, that represented the fraction of worms which still lived after certain time. Control worms were exposure to Cr(VI) at 10 mM, but without the treatment of AWX. * and ※ *P* < 0.05, ** and ## *P* < 0.01, ### *P* <0.001 compared to control

To investigate the protective effect of AWX against heavy metal Cr(VI) toxicity in *C. elegans*, chromium stress assay was used. As shown in Fig. 3, the survival fraction of the N2 adults' worm received AWX at o.1 was significantly higher than the control without treatment of AWX after 18 h (p < 0.01), 22.5 h (p < 0.01) and 24 h (p < 0.001) incubation. The similar effects of AWX at 0.083 and 0.125 were also seen, but not as strong as AWX at 0.1. The effects of AWX at 0.05 and 0.067 were not significant compared to the control. The significant enhanced effect at 24 h disappeared in the groups of AWX at 0.083 and 0.125, and was only observed in the group of AWX at 0.1. Moreover, according to the data in Fig. 2, most (about 91.0 %) of worms without any treatments were alive at 23 h, but about only 39.2 % of worms in the control to be exposure to Cr(VI) survived at near this time point (22.5 h) (seen the Fig. 3). In contrast, AWX at o.1 significantly elevated the fraction of survival worms roughly to 75.6 %.

### The protective effect of AWX was DAF-16-dependent

**Fig. 4:**
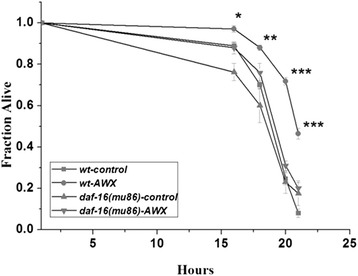
The protective effect of AWX was not shown in the mutational worm, *daf-16 (mu86)*. The meanings of X-axis and Y-axis are the same as ones in Fig. 2. Control worms were exposure to Cr(VI) at 10 mM, but without the treatment of AWX. AWX concentration was at 0.1 volume of AWX/total volume. * *P* < 0.05; ** *P* < 0.01; *** *P* <0.001 compared to wt-control. *wt* wild-type

**Fig. 5:**
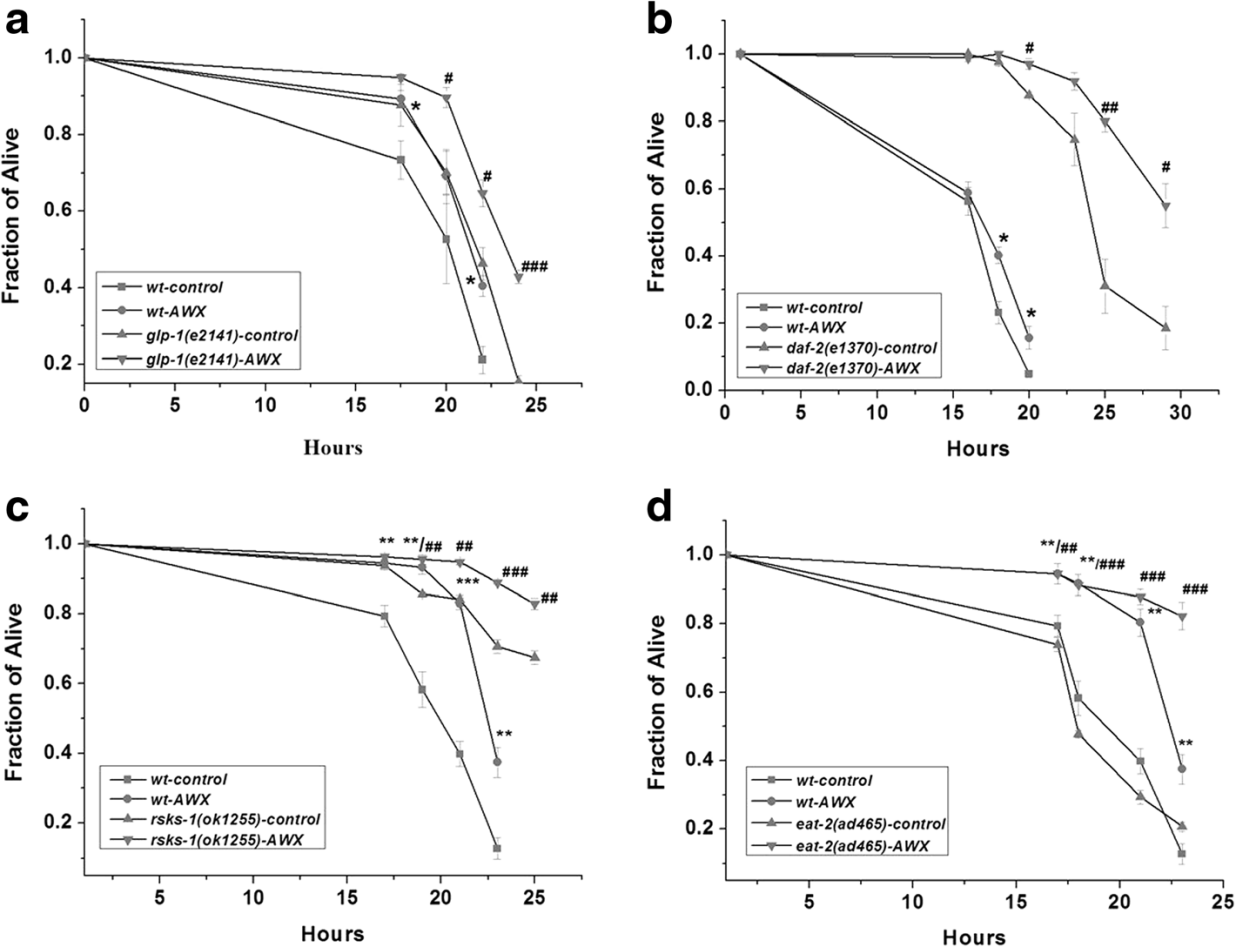
The protective effect of AWX was shown in the mutational worms, *glp-1(e2141)* (**a**), *daf-2(e1370)* (**b**), *rsks-1(ok1255)* (**c**) and *eat-2(ad465)* (**d**). The meanings of X-axis and Y-axis are the same as ones in Fig.2. Control worms were exposure to Cr (VI) at 10 mM, but without the treatment of AWX. AWX concentration was at 0.1 volume of AWX/total volume. * P < 0.05, ** P < 0.01, *** P < 0.001 compared to wt-control; # P < 0.05, ## P < 0.01, ### P < 0.001 compared to mutant-control respectively. wt, wild-type

For further researches on the action of AWX, several of characterized mutant nematode lines were used. As Fig. 4 showed, AWX at 0.1 had significant protective effects on the wild-type worms after 16 h (p < 0.05), 18 h (p < 0.01), 20 h (p < 0.001) and 21 h (p < 0.001) incubation, but no significant influence on the mutational worm, *daf-16 (mu86)*, implying that the DAF-16 function was essential for the action of AWX. However, experiments with other related mutant lines of worms, including *glp-1(e2141)*, *daf-2(e1370)*, *rsks-1(ok1255)* and *eat-2(ad465)*, demonstrated protective effects of AWX as same as in the wild-type worms (seen in Fig. 5), indicating that the protective effect of AWX was independent on the function of GLP-1, DAF-2, RSKS-1, and EAT-2.

### AWX reduced ROS production of *C. elegans* induced by Cr(VI) exposure in a time-dependent manner

**Fig. 6:**
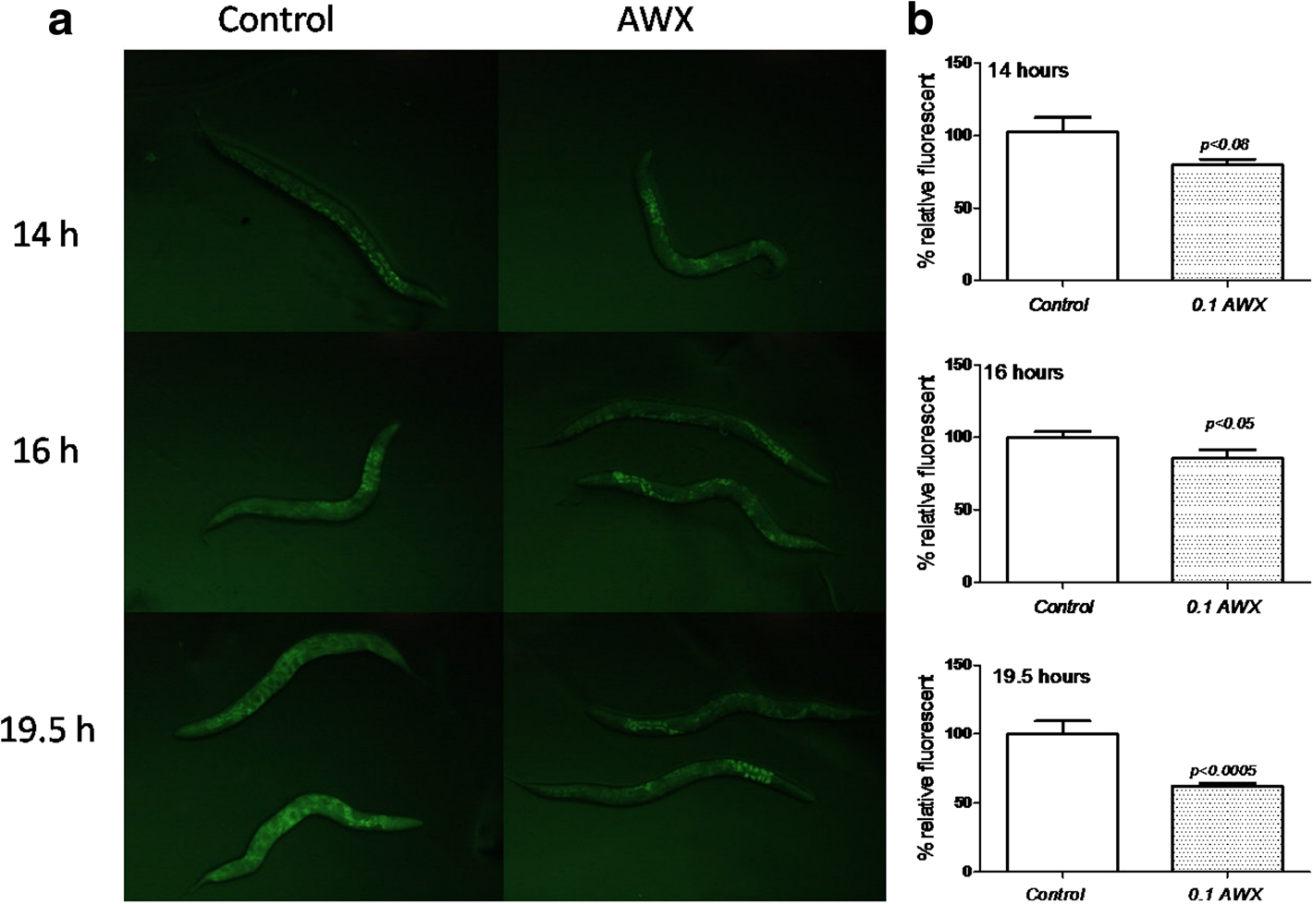
ROS production of nematodes exposed to Cr (VI). (**a**) Pictures showing the ROS production as detected by CM-H2DCFDA labeling in nematodes exposed to Cr (VI) with or without AWX treatment. (**b**) Quantitative comparison of ROS production in nematodes exposed to Cr (VI) with or without AWX treatment. Control worms were exposure to Cr (VI) at 10 mM, but without AWX treatment. AWX concentration was at 0.1 volume of AWX/total volume

Chromium exposure increased the ROS production of nematodes [7]. To quantify whether AWX treatment decreases ROS levels elevated by Cr(VI) exposure in *C. elegans*, the ROS production was assayed.As shown in Fig. 6, treatment of AWX at 0.1 significantly compromised the density of fluorescent or the elevation of ROS production after 16 h and 19.5 h (p < 0.05 and *p* < 0.0005, respectively). Although the decreased effect was not significant at 14 h (*p* < 0.08), the effect of AWX appeared to begin early, and became stronger with the extension of incubation time, to some degree.

## Discussion

The toxicity of Cr(VI) for *C. elegans* is well documented [7–9, 12, 13]. AWX (a traditional Uyghur medicine prescription), for the first time, was studied for the treatment of Cr(VI) intoxication in *C. elegans.* We, using endpoints of lethality, found that AWX at moderate contents (0.083, 0.1, 0.125 volume of AWX/total volume) had protective effects against the toxicity of Cr(VI) in *C. elegans*, although AWX at higher concentrations (more than or equal to 0.1 volume of AWX/total volume) was toxic*.*

The mechanistic cytotoxicity of Cr(VI) is not completely understand, however, a large number of studies demonstrated that Cr(VI) induces oxidative stress, DNA damage, apoptotic cell death and altered gene expression [5]. Concentration and time-dependent effects of Cr(VI) were demonstrated on increased ROS production and subsequent lipid peroxidation, enhanced excretion of urinary lipid metabolites, DNA fragmentation and apoptotic cell death in both *in vitro* and *in vivo* models [5]. According to our preliminary analysis regarding to the components of AWX, it contains chebulic acid, gallic acid, protocatechuic acid, and eugenol. Without exception, all of these compounds are potent antioxidants, by reviewing the literatures [14–18]. Moreover, considering that the AWX showed anti-oxidative effects in the previous studies in rats [3, 4], we supposed that AWX might protect the *C. elegans* from the oxidative damage induced by the Cr(VI). The experiment of ROS production demonstrated that AWX significantly reduced ROS production of *C. elegans* induced by Cr(VI) exposure. This result supports our hypothesis. The further indirect evidence comes from the studies used several mutant nematode strains.

The effect of AWX was dependent on the DAF-16 function. The DAF-16 is a forkhead transcription factor, which integrates signals from multiple pathways and regulates its downstream target genes to control diverse processes [19, 20]. It is an important signal transducer of the insulin/IGF-1 signaling pathway, and it also receives input from several other pathways that regulate life span and the germline [19, 20]. Our further experiments demonstrated that the protective effect of AWX was independent on the functions of DAF-2 (insulin-IGF receptor), GLP-1(notch, regulating self-renewal and differentiation of germ stem cells), RSKS-1 (worm homolog of mammalian p70S6K, promoting cell cycle progression in the germ line) and EAT-2 (involving in life span control), implying that the insulin/IGF-1 pathway and pathways regulating life span (through diet restriction) and germline were not involved [21–24]. Therefore, the further pathway of AWX affecting on DAF-16 is not clear yet, and awaits further studies. While DAF-16 translocates into the nucleus, it binds dozens of target promoters of genes directly, and acts as an activator or a repressor of transcription. These genes participate in stress protection, the promotion or prevention of longevity, dauer formation/maintenance and fat storage [19, 20]. DAF-16 activates stress-response genes. The microarray and/or the bioinformatics studies disclosed that antioxidant genes (such as superoxide dismutase, metallothioneine, catalase, and glutathione *S*-transferase), and small heat shock protein genes are involved in the protective effects in worms [19, 20]. Therefore, the intensive researches of these downstream target genes of DAF-16 should be helpful to elucidate the molecular mechanisms of AWX.

## Conclusions

In conclusion, AWX, a traditional Uyghur medicine prescription, had protective effects against the toxicity of Cr(VI) in *C. elegans.* As AWX suppressed ROS production of *C. elegans* induced by Cr(VI) exposure, and the protection was dependent on the DAF-16 function, the oxidative stress protective mechanism in worms should be involved.
